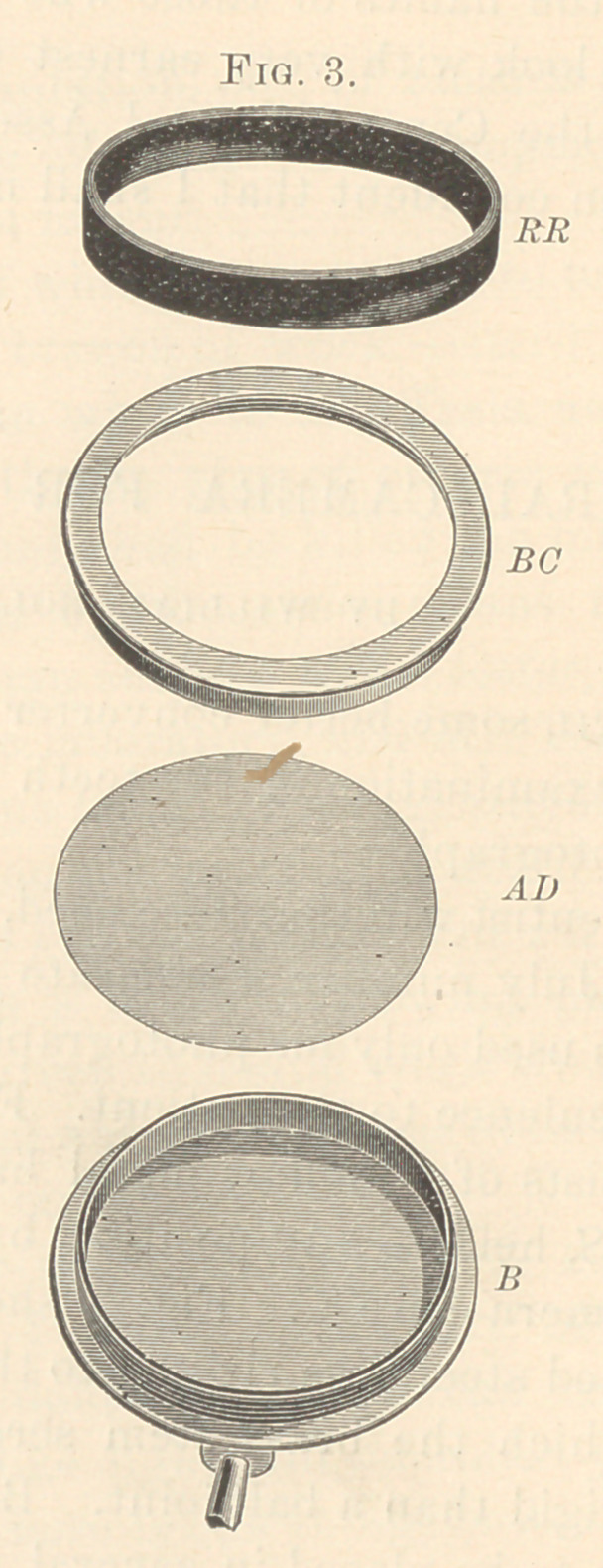# An Oral Camera for Röntgen Photography

**Published:** 1896-08

**Authors:** William Rollins

**Affiliations:** Boston, Mass.


### AN ORAL CAMERA FOR RÖNTGEN PHOTOGRAPHY.

BY WILLIAM ROLLINS, BOSTON, MASS.

UNTIL some better converter is found for the Röntgen rays, the visual examination of the teeth will give less perfect results than the photographic.

A dentist will therefore need, in addition to the converter shown in the July number, a separate camera, because where the instrument is used only for photography it may be smaller, and cause less inconvenience to the patient. Fig. 1 shows such a camera full size. It consists of a hollow metal handle, *MH*, a flexible sliding brass rod, *BS*, held in any position by the set screw *SC*, and supporting the camera-cell *CC*. Fig. 2 shows the back of the camera. The hardened steel boss riveted to the camera-cell has a threaded cavity into which the brass stem screws. This is simpler, smaller, and more rigid than a ball-joint. Bending the brass stem allows the camera to be placed in several positions. When this stem breaks

FIG. 1.

FIG. 2.

FIG. 3.

from frequent bending a new one can be inserted in a moment, the thread in the steel boss cutting a thread on the brass as it is turned in. Fig. 3 shows the camera in pieces. *RR* is a soft rubber ring, clasping the collar *BC*, when in position, as shown in Fig. 1, thereby preventing painful pressure on the mucous membrane. *AD* is an aluminum disk, which, when the brass collar *BC* is screwed on *B*, closes the camera light and water tight.

The instrument is to be used like the one described in the July number. A very simple form of camera can be made in the following way: Cut the sensitive films from kodak tissue, making them seven-eighths of an inch wide and an inch and a quarter long. Arrange them, after rounding the corners like the leaves of a book, and slip into a little envelope of black paper, and enclose them in a rubber cot, closing the end by folding over and securing with a bent steel wire. This sort of camera is very flexible, and is easily held against the mucous membrane with the finger. Whatever form of instrument is used, the best results are obtained by getting the sensitive photographic surface as near the point to be photographed as possible, and using that form of tube in which the rays are given off from a small intense source.

The uses to which Röntgen's discovery can be put in dentistry are many. It makes at least one new operation easy. Where a temporary tooth has remained for several years after it should have been shed I have always felt in doubt about the best course to take when it was a front tooth, because it was impossible to tell whether there was a second tooth under it.

Röntgen photography solves this problem, and enables us to remove the first tooth and open the socket of the second tooth to allow it to erupt.

In the September number of the journal I hope to describe and figure some of the simple generators I have devised for producing the electricity for lighting the tubes.

## Figures and Tables

**Fig. 1. f1:**
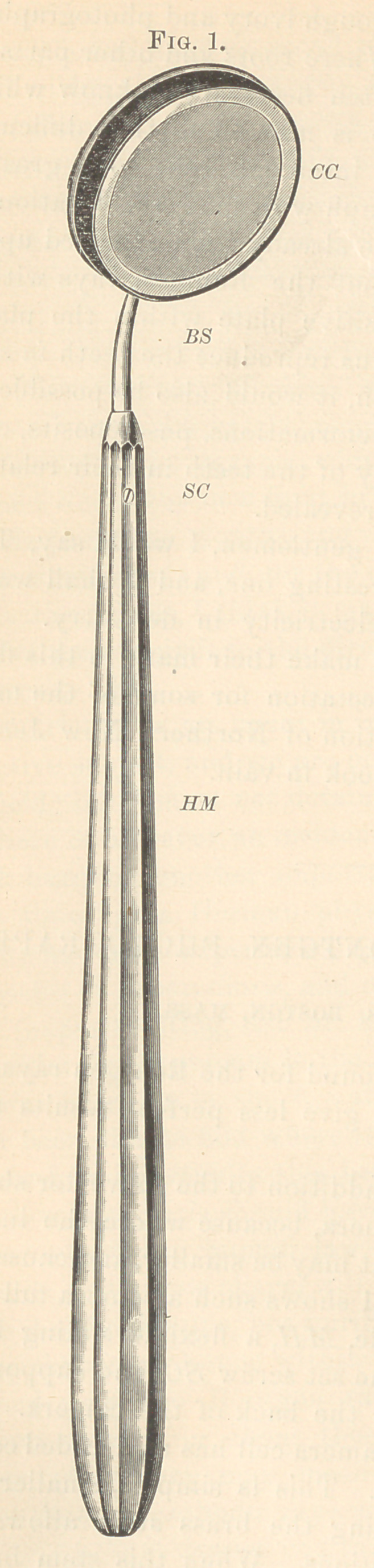


**Fig. 2. f2:**
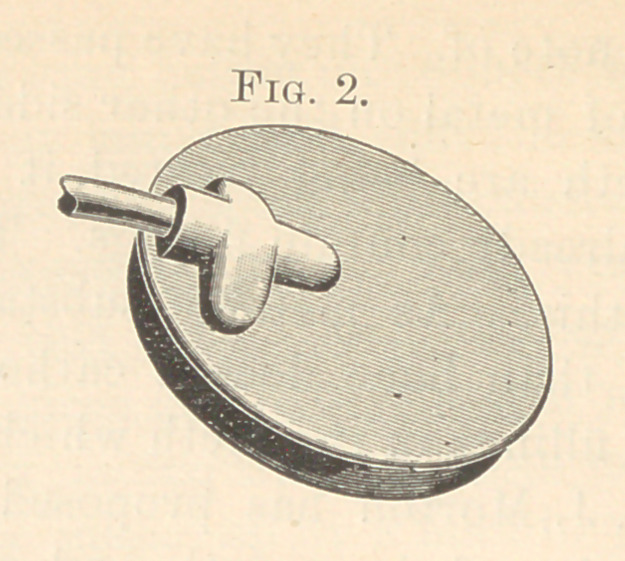


**Fig. 3. f3:**